# Internal Urethrotomy with Report of Cases

**Published:** 1888-12

**Authors:** W. S. Elkin

**Affiliations:** Atlanta, Ga., Lecturer on Genito-Urinary Surgery and Venereal Diseases, Southern Medical College


VOL. V. DECEMBER, 1888. No. 10.

### Original Communications.

### INTERNAL URETHROTOMY WITH REPORT OF CASES.*

BY W. S. ELKIN, M. D., ATLANTA, GA.,

Lecturer on Genito-Urinary Surgery and Venereal Diseases, Southern Medical College.

*Case 1.*—M. M., single, age 29; examination revealed three strictures, cause gonorrhœa. Two of large calibre in the pendulous portion of the urethra 1½ and 4 inches from meatus, and the third in the membranous portion of the canal that would only admit of a small filiform.

The posterior one was first divided with a Maisonneuve's instrument and the others were cut with Otis' dilating urethretome to thirty-four m. m. the normal size of urethra. The introduction of sounds was kept up for two months and patient discharged cured. The urethra was examined one year afterwards and no recontraction found.

*Read before Atlanta Society of Medicine, October, 1888.

*Case 2.*—E. E. L., age 33, widower; no history of gonorrhœa, but acknowledged the practice of masturbation while a youth. Two strictures, situated 2½ and 4½ inches from meatus, admitting a 26 bulb, were detected. April, 1884, a 4 per cent. solution of cocaine was used and the strictures divided by Otis' urethretome to the natural size of urethra 36 m. m. I have examined this patient recently and detected no recontraction.

*Case 3.*—R. B. W., age 26, single; cause, gonorrhœa; size of meatus 26 calibre, urethra 34. One stricture 3½inches back, size 24, performed internal urethrotomy, November 18, 1884. Have not since examined this case, but from letters received recently he assured me he is perfectly well.

*Case 4.*—C. McH., colored, married, age 35; cause of stricture, gonorrhœa; operated on January 25th, 1885, for a stricture five inches from meatus; would admit 22 bulb, cut to 32 m. m.

*Case 5.*—C. W., age 25, single; two strictures 1½ and 2 inches from meatus, admit 24. Operated on January 30, 1885, and stricture divided to 34; no recontraction to date. Cause of stricture, gonorrhœa.

*Case 6.*—Mr. P., married, age 35; three strictures 1½-3-5 inches, would admit 27 bulb. Divided to 34 m. m., October, 1886. Recontracted and patient again operated on April, 1887, and strictures cut to 36 m. m. Four months later patient was examined and found perfectly well.

*Case 7.*—M. B., age 69, married; stricture 5½ inches from meatus—only admit filiform. Cause of stricture, gonorrhœa contracted during the war. Operated on July 17, 1886, and stricture divided first with Maisonneuve's instrument and then with Otis' dilating urethretome up to 32 m. m. Patient died ten days later from acute nephritis. The health of this patient was being rapidly undermined and the operation was performed as a dernier resort. I believe now that the patient's chances would have been more favorable had I made a perineal section, as I now do in all cases of stricture situated in this locality if they cannot be cured by gradual dilation.

*Case 8.*—A. A. R., single, age 32; stricture 2½ inches from meatus, admit 26 bulb; divided June, 1886, to 34 m. m. Patient made a rapid recovery. Cause of stricture, gonorrhœa. No recontraction one year later.

*Case 9.*—L. R., married, age 38; three strictures, 1, 2¼, 4 inches from meatus. Would admit 28 bulb; operated on November 30, 1886, and cut to 32 m. m. Patient lives in an adjoining county, but has recently informed me that he is entirely cured.

*Case 10.*—C. A. R., age 23; stricture 3 inches from meatus, size 20 m. m.; no history of gonorrhœa; caused in infancy by a careless nurse running a safety pin through the organ. An urethral discharge set up at the time which lasted several weeks, and more or less irritation had been present until internal urethrotomy was performed, February, 1887, which resulted in complete relief of all these symptoms.

*Case 11.*—F. M., single, age 24; one stricture 3½ inches from meatus; size, 28 m. m.; cause gonorrhœa contracted two years previous; operated on April 23, 1887, and strictures divided to 33 m. m. Complete recovery.

*Case 12.*—G. H., age 25, single; two strictures 2½ and 4½ inches from meatus; very irritable but of large calibre, admitting readily 29 bulb sound, complicated by vesical irritation. Strictures divided April 30, 1887, to 35 m. m. Rapid recovery and with no recontraction up to date. Cause, previous attack of gonorrhœa.

*Case 13.*—M. G., age 27, single; one stricture; size, 30 m. m., near the bulb; very irritable and attended by a slight discharge. No history of gonorrhœa, but admitted practicing onanism to excess from sixteen to twenty years of age. Claimed to be impotent, though admitted that he frequently had nocturnal emissions; great mental distress. Operation performed May 1887, and stricture cut to 34 m. m. He now claims to be perfectly well with restoration of full and natural power to the genito-urinary organs.

*Case 14.*—C. D. R., single, age 24; four strictures 1, 2½, 3½ and 4½ inches from meatus, admitting 18 bulb, gradually dilated to 26 with sounds, and cut June 1, 1887, to 32 m. m.

Slight downward curvature of the penis for six months after the operation; cause of stricture gonorrhœa.

*Case 15.*—W. C. P., single, age 25; two strictures 2½ and 3½ inches from meatus; very irritable and attended with quite a copious discharge from the urethra; admit bulb 27; operated on September 1, 1887, and stricture divided to 32 m. m. The discharge of pus already existing irritated the cuts and considerable inflammation followed the operation. This lasted several weeks, after which it subsided and the patient made a good recovery. The introduction of the sound in this case was continued from time to time for four months.

*Case 16.*—H. J., single, age 27; strictures 1½, 3 and 4½ inches from meatus; quite irritable and attended with much discharge and pain on urination; admit 25 bulb; cause, a severe case of gonorrhœa contracted six months previous; operation performed November 4, 1887, followed by an increased discharge showing that the incisions had become inflamed. This rapidly disappeared although there was more or less curvature of the organ for two months after the operation. The introduction of sounds was kept up at varying intervals for three months and the patient discharged cured.

*Case 17.*—J. C. W., single, age 26; two strictures 2 and 4 inches from meatus; admit 29 bulb; very irritable, with slight discharge from penis. There also existed at the time a chronic case of ammomical cystitis of more than two years duration. Stricture divided to 36 m. m. January. 15, 1888. The patient made a rapid recovery and was discharged in two months cured.

*Case 18.*—A. B., colored, age 35; three strictures 1½, 3½, 5½ inches from meatus. This case had been operated upon six months before, but as all the fibrous bands had not been divided there was recontraction. He also suffered with marked curvaature of the penis and considerable irritation of urethra attended by discharge. Strictures re-divided February 10th, 1888, to 38 m.m. before the class at the Southern Medical College. Discharge ceased, and the curvature of the organ is gradually being relieved.

*Case 19.*—C. C. C., age 29, single; one stricture 3½ inches from meatus; cause, gonorrhœa contracted six months previous.

Slight discharge from meatus and the whole canal more or less sensitive; admit 29 bulb; operated on March 15, 1888, and cut to 34 m. m. Patient made a rapid and complete recovery and was married in one month after operation. Six months later examination revealed no recontraction.

*Case 20.*—B. B., medical student, age 25, single; one stricture at bulb, large calibre, admitting 30 m. m., complicated, with vesical irritation. Cause, gonorrhœa. Stricture cut March 22, 1888, to 35 m. m. Marked relief of vesical symptoms followed, but as the patient left the city a few days after the operation, I am not able to report the result.

*Case 21.*—H. M., colored, age 38, married. One stricture four inches from meatus; admit 28 bulb; cause, gonorrhœa. Operated on March 23, 1888. Discharged cured two months later.

*Case 22.*—J. C. W., medical student, married, aged 32. Two strictures, 2½ and 4½ inches from meatus. No discharge, but quite irritable and attended by frequent micturition. Admit 29 bulb. Cause, gonorrhœa, contracted six years previous. Operated upon July 21, 1888, and stricture divided to 36 m. m. Patient discharged cured six months later.

*Case 23.*—F. B. G., single, age 27. Two strictures, 1 and 3½ inches back. Complicated with a severe case of cystitis of more than two months' duration. Cause, gonorrhœa, contracted four months previous. Admit bulb 25. Operated on October 8, 1888, with immediate relief of vesical troubles. I am still introducing the instruments in this case, but the patient reports himself as perfectly cured.

In all these cases, the meatus was smaller than the normal calibre of the urethra, and was divided with a straight, blunt-pointed bistoury, before the operation for the division of the strictures was begun. Otis' dilating urethretome was used in dividing the stricture in the pendulous portion of the canal.

Cases 2–17 are undoubted illustrations of strictures resulting from masturbation, and 7 the result of a traumatic injury. The others were caused by previous attacks of gonorrhœa.

The division of strictures confined in the pendulous portion of the urethra, is a comparatively safe operation, and altogether satisfactory if properly performed. If the strictures are attended with much discharge, this should always be reduced as much as possible before the operation, or else the cuts become inflamed, and the recovery is not so rapid. In all the cases operated upon by me where there was considerable discharge, the downward curvature of the penis was most marked, and required a much longer time for the relief of this deformity.

Strictures situated in the membranous portion of the urethra should be gradually dilated, or, if this is not possible, divided by means of external urethrotomy. I do not believe that strictures in the pendulous portion of the canal can be radically cured by a gradual dilatation, yet we are often compelled to adopt this means, since many patients object to the cutting operation.

